# Do windy areas have more wind turbines: An empirical analysis of wind installed capacity in Native tribal nations

**DOI:** 10.1371/journal.pone.0261752

**Published:** 2022-02-25

**Authors:** Laura E. Evans, Nives Dolšak, Aseem Prakash

**Affiliations:** 1 Evans School of Public Policy and Governance, University of Washington, Seattle, Washington, United States of America; 2 School of Marine and Environmental Affairs, University of Washington, Seattle, Washington, United States of America; 3 Department of Political Science, University of Washington, Seattle, Washington, United States of America; Universiti Sains Malaysia (USM), MALAYSIA

## Abstract

The decarbonization of the electricity sector is leading to a substantial increase in the demand for wind energy. Will tribal nations, which account for 7.8% of utility-scale wind capacity, benefit from this policy shift? To examine why tribal nations vary in translating wind energy potential into wind installed capacity, we have constructed an original dataset of the potential as well as the location of wind turbines across tribal nations. Our statistical analysis of 286 tribal nations suggests that wind energy potential is *not associated* with wind installed capacity. Instead, casino square footage, a proxy for tribal nation’s administrative capacity and business acumen, is associated with wind installed capacity. Political orientation plays a role as well: tribal nations are more likely to have wind installed capacity when they value tribal sovereignty. While tribes suffering from natural disasters do not install more wind turbines, those receiving federal grants for wind energy projects, and located in states that already have a substantial number of wind turbines, are more apt to have wind turbines. Surprisingly, tribes located in states with renewable portfolio standards *do not* show an association with installed wind turbines capacity.

## Introduction

What explains variations in wind electricity generation capacity in Native American tribal lands? Specifically, why do tribal nations vary in translating wind energy potential into wind installed capacity? The Seneca Nation’s installation of a wind turbine in western New York illustrates a success story. After receiving three grants from the Department of Energy’s Office of Indian Energy Policy and Programs (in 2003, 2007, and 2014), Seneca Nation installed a 1.7-megawatt wind turbine that significantly increased the availability of electricity and decreased cost for some of its members [1]. Yet, scholars have also noted the “paradox of plenty” where tribal nations with rich potential for renewables have not exploited these resources due to cultural and sovereignty issues [2] and legal obstacles [3]. Take the case of the Crow Tribe, which has made significant efforts in the pursuit of wind power development, but challenges of land status and securing outside investors have obstructed the realization of those objectives [4].

One of the remarkable developments in the energy sector is the decline of coal in electricity generation and its replacement by natural gas and renewable energy, including wind. Across the United States, electric utilities are declaring their intentions to fully convert to renewable energy, often in response to mandates such as renewable portfolio standards. Many state governments have announced plans to decarbonize their electricity grids over the next two decades completely. In addition, many companies such as Amazon, Microsoft, and Google that have energy-intensive cloud computing businesses have signed long-term contracts for the purchase of renewable energy for their facilities. Native American tribal governments are also embracing renewable energy even though many have substantial fossil fuel deposits. For example, although the Navajo Nation derived substantial revenue and employment from coal power generation, with the closure of this facility, it is increasing its investments in solar energy [5].

Investments in renewable energy can help Native American tribes increase electricity availability in a sustainable way, increase autonomy, and provide new revenue sources because much of the electricity will be sold to non-tribal customers. This paper examines variations in the installation of wind energy capacity by 286 Native American tribal nations located in 32 states. Our analysis excludes Hawaii, Alaska, and Oklahoma, for reasons we discuss later in the paper.

Many tribes are located in areas with high wind power potential. According to the analysis conducted by the National Renewable Energy Laboratory (NREL), tribal lands that represent 5.8% of the land area in the contiguous United States have substantial renewable energy technical potential, including 7.8% for utility-scale wind [6]. The Intertribal Council on Utility Policy assessed that Great Plains tribes could supply energy to 50 million homes based on wind energy alone [7]. Indeed, Bronin [8] suggests that there is sufficient renewable energy potential on tribal land to meet the electricity needs of the entire United States. Thus, the key variable of interest is the technical wind potential. All wind turbines in the U.S. require Federal Aviation Administration (FAA) approval. We use FAA’s Obstruction Analysis/ Airport Airspace Analysis (OE/AAA) dataset to access the precise location of all built turbines.

Yet, potential does not necessarily lead to installed capacity and production. Of 2,343 trillion BTU of wind energy consumed nationwide, NREL estimates just 237 million BTU were generated on tribal lands, which is only 0.02% of the wind consumption! [9] What hampers the conversion of the technical potential into installed wind capacity? Why are private developers not working with tribal governments? Is there insufficient federal help? To develop business partnerships with private energy companies or to access federal resources, tribal nations must have the business acumen and administrative capacities [10, 11] to navigate issues such as the transferability of renewable energy tax credit, an inability to capitalize on the Federal Production Tax Credit and double taxation in tribal and state jurisdictions [12–14]. More broadly, a switch from a fossil fuels-based economy to renewables requires an administrative support system with low transaction costs because the political and economic underpinnings of these new technologies are still evolving.

Our results suggest that the technical potential does *not have* a statistically significant association with tribes’ installed wind capacity. This is both good news and bad news. On the one hand, it shows how Native American tribes have yet to convert the technical potential into a viable economic asset. This is worrisome given both economic poverty and energy poverty in many Native nations [15], an issue observed in other countries as well [16]. On the positive side, it suggests that a stronger policy push and incentives for decarbonization could create substantial economic benefits for tribes. Because wind is not an exhaustible resource (unlike, say coal mines) and does not cause local air pollution, renewable energy generation could provide a sustained income stream to Native Nations. And when renewable energy replaces fossil fuel capacity, as in the case of the Navajo Nation, it could create local health benefits of cleaner air.

We also find that renewable portfolio standards (RPS) in the neighboring states, a key driver of renewable energy, *is not* associated with tribal wind capacity. This is surprising because RPS (even after accounting for their varying stringency) create the demand for renewable energy that tribal nations are in the position to satisfy [17–19]. What then hampers their ability to convert renewable energy demand and technical wind potential into installed capacity. We suggest the important role of administrative capacity and business experience. This is probably why we find a statistically significant association between casino footage and wind capacity. Casinos (which are owned by tribal governments) provide tribes with experience of dealing with non-tribal business partners and navigating administrative complexities. Akee et al. [20] note:

Tribal governments have also used the revenues from gaming to fund other economic development, based on the widely shared view that Indian gaming will not provide sustained economic growth indefinitely. Typically, the pattern begins with developing adjacent hotels, conference halls, amphitheaters, and other amenities that increase the drawing power and visit durations of gaming facilities…. Finally, they turn toward more distinct sectors… often redeploying the management experience gained in tribal gaming development.

## Data

We have constructed an original database of wind installations drawing data from multiple sources. For our dependent variable, the presence of wind turbines (henceforth wind installed capacity), we use Federal Aviation Administration (FAA) data on built wind turbines from 1995 to January 2021. All wind turbines in the U.S. require an FAA permit, which precisely documents the location of the turbine. FAA shares the resulting Obstruction Analysis / Airport Airspace Analysis (OE/AAA) dataset with natural resource management agencies, which the U.S. Fish and Wildlife Service’s Ecological Services posts in a somewhat more user-friendly format than the FAA does [21]. To identify turbines on federally recognized tribal lands, we overlay FAA turbine location data with the Bureau of Indian Affairs’ American Indian and Alaska Native Land Area Representation (AIAN-LAR) Geographic Information System dataset [22]. AIAN-LAR includes all tribes that have a land base. Tribes that have federal recognition but without a land base are excluded from AIAN-LAR.

Our analysis excludes Hawaii, Alaska, and Oklahoma because of the unique legal circumstances in each state. Tribes in Hawaii do not have federal recognition. While tribal governments in Alaska have much in common with tribal governments in the rest of the U.S., the Alaska Native Claims Settlement Act created important, distinct features of Alaska Native governments. The unique history of Oklahoma has produced a unique landscape of tribal land status. As a result, we were unable to identify whether turbines in Oklahoma were on or off tribal lands [23].

### Independent variables

We examine the role of technical wind potential, institutional factors (business acumen and administrative capacity as proxied by tribal casinos, Department of Energy support through the wind energy grants), and the political environment (RPS and concerns about tribal sovereignty) in which the tribes are located. The key variable of interest is the tribe’s *technical potential for wind power* at the utility-scale (there is very little distributed scale, below 1 megawatt, wind power). To harness wind energy for electricity generation, the tribal land must have sufficient wind flow or technical potential. Therefore, we expect that tribes with more renewable energy potential are more apt to pursue renewable energy projects. These data are drawn from the Tribal Energy Atlas, which researchers at the National Renewable Energy Laboratory (NREL) developed to provide geospatial data on assessed technical renewable energy potential. The applied methodologies are provided in Milbrandt et al. [24] Energy potential may not automatically translate into energy generation capacity—institutions and expertise matter. Actors must have the economic and business expertise to recruit outside investors, select appropriate technologies, and oversee the construction project. We do not control for solar energy in our model. The reason is that to date, only 4 tribes have installed utility-level solar projects. Hence, there is insufficient variation on this variable for it to be included in our model. We expect tribes with business experience have an advantage in this regard. Hence, we consider the *size of tribal casinos* logged. Revenues from specific casinos are not publicly available, as they are proprietary information. Following Walker and Jackson [25], we consider the square footage of tribally-owned casinos as a proxy for casino revenue [26]. The average gaming operation is 71,151 square feet. Thirty-five percent of tribal governments in the sample do not have a casino.

Arguably, support for wind energy might reflect the desire for energy self-sufficiency and energy sovereignty [27] and not directly related to climate change. Thus, we also consider the tribes that were members of CERT (Council of Energy Resource Tribes) before it disbanded in 2012 [28]. CERT advocated for tribal energy independence and economic independence, but it was indifferent as to what forms of energy development tribes used. For example, some members had a lot of coal mining, while others were pursuing renewables. In addition to CERT, our model also controls the presence of fossil fuel extraction on tribal lands. The intuition is that a high salience of this industry might create a pressure group that dissuades tribal government from embarking on wind projects, which eventually seek to phase out fossil fuel from electricity generation. An alternative expectation, however, is that tribes involved in fossil fuel extraction might develop the business experience that would then aid wind projects.

Most tribes do not have experience with renewable technologies and need external support to build on them. Hence, we control whether the tribe has received a *Department of Energy’s grant* for developing wind power. The U.S. Department of Energy’s Office of Indian Energy Policy and programs has been funding energy-related projects on tribal lands since 2010 [29]. A predecessor program, the Energy Efficiency and Renewable Energy’s (EERE) Tribal Energy Program was providing funding prior to 2009 [30]. These projects have helped tribal governments build the institutional capacity to manage their energy needs and to assess the feasibility of renewable energy technologies. Some tribes in our sample have renewable energy projects and programs that are funded by this grant right now or that were jumpstarted by this grant in earlier years. We include a binary variable with 1 denoting a tribe that has secured at least one DOE grant and 0 denoting a tribe that did not receive any DOE grants.

Two state-level factors could also influence wind installed capacity. A large number of U.S. states have adopted renewable portfolio standards (RPS) which obligate utilities to source a share of their electricity from renewable sources such as wind and solar. There is literature debating the extent to which RPS drives wind installed capacity [31, 32]. In terms of mechanisms, scholars note that the RPS might depend on wind energy potential [33]. Tribal nations do not mandate RPS. Typically, they are connected with the state grid, which could drive demand for wind energy generation on tribal lands. But sometimes, the state grid itself is connected to a regional grid which means that a tribal wind turbine could provide renewable electricity to the state, which is not contiguous to the tribal land. Recognizing the diversity in RPS design [34], we rated state RPS according to two features of the standard: the maximum level of zero-emissions energy mandated and the number of years after 2000 before the level must be achieved. New York, with a requirement for 100% zero-emissions energy by 2040, received a score of 2.5. States with no RPS received a score of zero. The data are from the National Conference of State Legislators [35].

Second, we also control for the *density of built turbines* in the state. There may be multiple effects on tribes from being located in regions where the wind energy sector is thriving. A high density of turbines reflects a windy climate, but it also indicates political and economic conditions. Wind turbines can proliferate if state and local land-use regulators treat wind energy favorably. We use OE/AAA data to identify all built turbines in each state (outside tribal areas), and we divide that number by the square miles in the state.

Scholars debate whether a direct experience with extreme weather events might influence individual-level or institutional responses to climate change. The logic is that climate mitigation is a global public good, although with local consequences. Thus, unless actors personally experience the costs of neglecting climate action, they might be unwilling to incur private costs to provide for climate mitigation through policies such as decarbonization of the electricity sector. Thus, our models include measures of tribes’ ecological conditions. In addition, we consider tribes’ *climate vulnerability*. We expect that climate disasters serve as focusing events. In places with fewer climate disasters, policy attention may drift away from climate change, given that native nations grapple with several pressing policy problems [36, 37]. We include the total number of tribal declarations of natural disasters by the Federal Emergency Management Agency (FEMA) from 1997 to May 2019 [38]. In Table 1, we present descriptive statistics for all variables in our analysis.

**Table 1:** Descriptive statistics.

*Variables*	*Mean*	*Standard Deviation*	*Minimum*	*Maximum*	*Frequency*
**Continuous**					
Number of turbines	0.66	7.49	0	122	-
Potential wind capacity	2,030	10,602	0	162,427	-
Casino size, logged	6.50	5.45	0	13.65	-
Natural disasters declarations	0.77	2.28	0	29	-
Built turbines per mi^2^ in state	71	52	0	445	-
Renewable Portfolio Standards	1.40	.96	0	2.5	-
Per capita income	$20,760	$19,468	$7,540	$228,683	-
% of households that are off grid	22.5	25.2	0	100	-
Weeks of drought	2.44	2.35	0	9.49	-
**Dichotomous**	-	-	-	-	
Fossil Fuel	-	-	-	-	11%
Has installed wind capacity	-	-	-	-	4%
Belonged to CERT	-	-	-	-	15%
DOE wind grant	-	-	-	-	9%
In big city	-	-	-	-	20%
Own EPA Office	-	-	-	-	28%

## Findings

We fit a negative binomial event count estimator given that our dependent variable is over-dispersed. Our main model specification is expressed as follows:

$$\mathrm{Pr}(Y=y_{i}|\mu_{i},\alpha )\frac{\Gamma (y_{i}+\alpha^{-1})}{\Gamma (\alpha^{-1})\Gamma (y_{i}+1)}{\left(\frac{1}{1+\alpha \mu_{i}}\right)}^{\alpha^{-1}}{\left(\frac{\alpha \mu_{i}}{1+\alpha \mu_{i}}\right)}^{y_{i}}$$

where $\mu_{i}=exp(\beta_{0}+\beta_{1}\chi_{1i}+\beta_{2}\chi_{2i}+\beta_{3}\chi_{3i}+\beta_{4}\chi_{4i}+\beta_{5}\chi_{5i}+\beta_{6}\chi_{6i}+\beta_{7}\chi_{7i}+\beta_{8}\chi_{8i})$ and $\alpha =\frac{1}{\nu }$ and *χ*_1_ = *potential wind capacity*, *χ*_2_ = *ln*(*casino size*), *χ*_3_ = *CERT*, *χ*_4_ = *fossil* fuel extraction on tribe’s lands, *χ*_5_ = DOE wind grant, *χ*_6_ = FEMA, $\chi_{7}=\frac{built\;turbines\;in\;state}{{miles}^{2}}$, *χ*_8_ = state Renewable Portfolio Standard

Table 2 presents regression analysis to examine variations in wind installed capacity across tribal nations. Our results hold even when exclude the Navajo Nation from the regression analysis because of its unusually high potential wind capacity: more than three times larger than that of any other tribe.

**Table 2:** Predictors of tribal wind turbines.

	Main Model
Potential wind capacity (by 100,000)	-1.10
	(5.53)
Casino size, logged	1.44***
	(.32)
CERT (Tribe belonged to CERT)	2.24**
	(.84)
Fossil fuel extraction on tribe’s lands	3.37***
	(1.02)
DOE wind grant	3.42***
	(1.31)
FEMA (Natural disasters declarations)	-.34*
	(.19)
Built turbines per mi^2^ in state	.067***
	(.015)
State Renewable Portfolio Standard	-.48
	(.47)
Constant	-25.56***
	(4.35)
N	286

Negative binomial regression, robust standard errors, excluding Oklahoma tribes, Alaska tribes, and the Navajo Nation. Standard errors in parentheses. Statistical significance at the 10%, 5%, and 1% level indicated by *, **, and ***, respectively.

The key finding is that tribal potential wind capacity is *not* statistically associated with wind installed capacity. This is a troubling finding because it suggests that Native Nations have not been able to exploit a valuable resource and leverage an important economic development opportunity that the rapid decarbonization of the electricity sector offers. Our finding is consistent with the scholarship that by itself, resource availability may not spur economic development: institutions and administrative capacity are required [39–41]. In our case, key institutions pertain to the business and administrative experience of the tribe.

How might tribal governments acquire such competencies? The role of the casino industry is crucial here. Creating utility-scale wind power facilities requires Native Nations to have substantial business acumen and administrative capacity to negotiate with non-tribal companies. Casinos provide this sort of experience to tribal governments, especially when they operate on a large scale, often alongside hotel facilities. More broadly, scholars note that the casino industry tends to positively affect economic growth in both tribal and non-tribal areas [42, 43]. Hence, for the purpose of this paper, casino square footage serves as a theoretically persuasive proxy for the business and administrative competence of the tribal government. Across our models, casino size (logged) has a positive, statistically significant association with wind installed capacity.

Tribes could acquire administrative capacity in other ways as well. We find that federal assistance for wind energy, which often allows tribal governments to hire individuals with technical skills, is associated with wind installed capacity. How do politics affect wind energy capacity? The quest for wind energy reflects the desire for energy self-sufficiency and sovereignty. Indeed, we find that tribes who joined CERT (Council of Energy Resource Tribes) are more likely to have wind turbines. This suggests that wind installed capacity reflects concerns about energy sovereignty as well. To our surprise, we find that the fossil fuel industry is positively correlated with wind energy. Arguably, experience with the fossil fuel industry might provide tribes with business acumen and administrative capacity to deal with non-tribal businesses interested in wind energy development.

State-level factors are also associated with installed wind capacity in unanticipated ways. Much to our surprise, the presence of RPS in the neighboring state is not statistically correlated with installed wind capacity. Recall, RPS was expected to create demand for renewable energy that would have motivated electricity companies to explore tribal lands for this purpose. But this demand side driver does not seem to be associated with wind installed capacity; the bottleneck seems to be on the supply side, administrative and business capacity.

Yet, we find that the density of turbines in this neighboring state has a positive, statistically significant association with turbine installation on tribal lands. Tribal wind development benefits from settings where the wind energy business sector is thriving, and state and local regulators have permitted this industry. This lends additional support to the argument that key impediments for wind installed capacity seem to be located within tribal nations, and not in the demand side of the renewable energy puzzle.

Prior experience with natural disasters is not associated with wind energy capacity. This suggests that commitment to climate mitigation via the decarbonization of the electricity sector might not be driven by prior experience with extreme weather events.

To evaluate the magnitude of effects, we calculate the incidence rate ratios for a standard deviation change in each continuous variable and a change from 0 to 1 for each dichotomous variable. Table 3 shows these ratios. The numbers should be interpreted carefully. Tribal leaders have often described, and scholars have already documented, the severe barriers economic development on tribal lands. Therefore, it should be no surprise that tribes find wind power development nearly impossible if they lack any particular advantages—no participation in CERT, no fossil fuel economy, no federal grants for wind power development, and typical values for continuous variables. Put simply, the typical tribe is locked out of wind power development, and the predicted number of turbines is .000007. Yet as advantages accrue, tribes have prospects of threading the needle. For a tribe with a casino one standard deviation above the mean size, that participated in CERT, and that has a fossil fuel economy, we predict 4.9 wind turbines. By far, the most significant contributor to that increase is the size of casinos.

**Table 3:** Incidence rate ratios.

Potential wind capacity (by 100,000)	.88
Casino size, logged	2390
CERT (Tribe belonged to CERT)	9.44
Fossil fuel extraction on tribe’s lands	29.00
DOE wind grant	30.57
FEMA (Natural disasters declarations)	.48
Built turbines per mi^2^ in state	30.81
State Renewable Portfolio Standard	.62

Calculated for a 1 standard deviation change in each continuous variable and a change from 0 to 1 for dichotomous variables.

### Robustness checks

We recognize that the size of a tribe’s casino might correlate with whether the casino is in a large metro area. Large metro areas might also be associated with features that aid wind energy development: better access to the electric grid, wealthier tribal members, and a denser local network of potential outside investors in tribal enterprises. Hence, in the robustness checks, we control for these dynamics. Specifically, we control for whether tribal lands are in a Metropolitan Statistical Area with more than one million residents, per the 2019 American Community Survey (ACS) by the U.S. Census Bureau.

This variable also correlates with per capita income on a tribe’s lands and the percent of households on tribal lands that heated their homes with off-grid energy such as wood, coal, coke, kerosene, or other liquid fuels. While the absence of an energy grid may be a rarity in most of the country, 22.5% of Native households on tribal lands heat their homes with wood, coal, coke, kerosene, or other liquid fuels. All three variables are significant, and our key results about technical wind capacity and casino square footage hold (Model 1).

Installing wind turbines might require the tribe to be familiar with the regulatory and permitting process. Thus, we considered whether tribal experience with running environmental programs would change our main effects. Specifically, we considered whether a tribe operated its own EPA office, rather than having the US EPA deliver services to the tribes, via the US EPA’s Treatment-As-State program. When we included this variable, the results from our main results remained unchanged (Model 2).

In our main model, we controlled for the tribal declarations of natural disasters to FEMA. Yet, this measure does not cover the full gamut of climate-related challenges that tribes might face. Specifically, these declarations do not cover drought. The reason is bureaucratic: funding to address drought comes from the U.S. Department of Agriculture (USDA). Thus, we include the average consecutive weeks of drought per year at levels that impose mandatory water use restrictions from 1980 to 2019. Data are reported in the EPA Climate Indicators study and gathered from the U.S. Drought Monitor. As the indicators frequently do not cover tribal areas, we used data for the nearest county. We ran a model that included drought and found the key results unchanged (Model 3). Table 4 presents the key results from our robustness checks.

**Table 4:** Robustness checks.

	*Model 1*	*Model 2*	*Model 3*
Potential wind capacity (by 100,000)	-3.01	.30	-3.72
	(7.26)	(6.20)	(5.50)
Casino size, logged	1.03***	1.46***	1.53***
	(.25)	(.44)	(.40)
Tribe belonged to CERT	3.85***	2.91***	1.93**
	(1.04)	(.91)	(.81)
Fossil fuel	2.94**	3.03***	3.70***
	(.82)	(1.16)	1.09)
DOE wind grant	2.54**	3.47**	3.56**
	(1.29)	(1.36)	(1.45)
Natural disasters declarations	-.31	-.41**	-.27
	(.19)	(.21)	(.18)
Built turbines per mi^2^ in state	.072***	.062***	.062***
	(.014)	(.015)	(.013)
State RPS	-2.15***	-.48	-.65
	(.61)	(.50)	(.55)
In a big city	5.80***		
	(1.40)		
Per capita income on tribe’s lands (in thousands)	-.015**		-26.13***
	(.0077)		(5.15)
% of households that are off grid	.10***		
	(.036)		
Own EPA Office		-1.24*	
		(.68)	
Drought (drought four)			1.01
			(1.10)
Constant	-22.48***	-24.79***	-26.13
	(4.10)	(5.22)	(5.15)
N	250	286	275

Negative binomial regression, robust standard errors, excluding Oklahoma tribes, Alaska tribes, and the Navajo Nation. Standard errors in parentheses. Statistical significance at the 10%, 5%, and 1% level indicated by *, **, and ***, respectively.

## Conclusion

Do resources bring about economic prosperity or do resources translate into economic gains only in the presence of appropriate administrative capacity and institutions to exploit them? We examine this question in the context of wind energy resources that Native Nations possess. The surprising finding is that wind energy potential has not translated into installed wind energy installed capacity. This suggests that tribal governments’ wind energy development is far more a social, political, and economic phenomenon than a physical one.

We find that tribal casinos, a proxy for the government’s administrative capacity and the experience the government has with setting up and running a commercial enterprise, are associated with wind energy installation. This suggests that that administrative capacity is the bottleneck to turn wind potential into installed capacity—the substantive and statistically significant effects of casino size support our speculation that richer jurisdictions tend to have stronger capacities to start wind energy projects, and most vitally, to push those projects to completion.

Our paper should not be interpreted as implying that tribal governments are slow to take advantage of renewable energy resources and convert them into commercially viable assets. Installing turbines is both costly and lengthy. It involves confronting many administrative hurdles. A turbine proposal is not a frivolous exercise; tribes must submit a great deal of specific data about their plans to the FAA. Tribes may seek FAA approval while they are still recruiting outside investors. Future research could explore why certain proposals get translated into actual turbines, even within the context of a given tribe, and explain the variation in the time from plan proposal to turbine installation. This analysis could add further nuance to the understanding of wind industry development in tribal lands.

Sometimes wind potential remains untapped because utilities find that wind facilities produce electricity intermittently, specifically, only when there is a sufficient wind flow. The problem is accentuated when there is a mispatch between electricity generation and peak electricity demand. For example, there might be demand surge in the evening when people return home, start their appliances and charge their cars. But if wind flows are feeble during the evening, there is excess demand for electricity. This means wind systems require back-ups to meet the peak demand, which often is provided by gas-fired turbines. Scholars note that the rapid technological improvements in battery storage could address this issue [44–46]. Wind turbines could generate electricity when the wind flow is strong and store the excess electricity in batteries. When electricity demand peaks, utilities could draw electricity from these batteries even when wind turbines might not be functioning. Future research should track how decreases in battery storage costs might help improve the economics of wind energy and therefore wind penetration in tribal areas.

Wind penetration is also impeded by limited transmission capacity, which impacts the economic viability of wind facilities. Of course, new transmission lines could be added but along with increased costs, this requires cumbersome regulatory processes, and sometimes dealing with local NIMBY dynamics. Scholars note that the development of a “smart grid” could relieve network congestion. Historically, transmission ratings (thermal ratings to ensure that lines do no overheat when they transmit electricity) tend to be based on fixed weather conditions. Because such weather conditions can change (such as temperature in lower than assumed in the calculations), the actual transmission capacity might remain underutilized. This also could lead to the idling of wind farms when they could be generating electricity and earning revenue. However, the development of new sensory technology such as dynamic thermal rating system can help relieve grid congestion [47, 48]. The idea is that instead of assuming a fixed transmission capacity for the line (which often based on conservative assumptions about weather), the actual capacity can be assessed by sensors that track local weather conditions. Whenever there is a gap between assessed capacity and the real capacity, wind facilities that might have been idled due to network congestion, can be activated. If tribal wind energy is impeded by network congestion issue, the development of smart grid technologies could help improve the uptake of wind energy in native tribal lands.

We acknowledge the literature on turbine location as an issue of climate justice and environmental racism. Climate justice has several dimensions, and an important one pertains to the asymmetrical distribution in the gains from decarbonization [49]. Typical examples include subsidies for electric cars or rooftop solar, which tend to flow to richer households. The inability of Native Nations to convert wind energy potential into a tangible economic resource raises questions as to why these nations are not benefiting from the broader thrust of decarbonizing electricity generation, which to some extent benefits from governmental subsidies. We recognize that wind turbines might be welcomed in all communities. Indeed, several municipalities and counties have enacted laws to discourage the installation of wind energy turbines [50]. Tribal nations, as sovereign governments, might decide that wind turbines are not a good fit for their lands and citizens. Eventually, the decision about building wind turbines must be grounded in local knowledge of needs and circumstances. Nevertheless, as countries and companies adopt net zero-emission targets, demand for wind energy will increase. This does provide an important economic opportunity for tribal nations to leverage their wind energy potential into a tangible economic resource.

Our paper has several limitations. First, we offer a cross-sectional analysis of wind turbine count, not a longitudinal one. As climate policy has evolved over the years, longitudinal analysis can add more nuance to how the policy environment might shape the evolution of the wind energy industry on tribal lands. Second, our dependent variable is the count of wind turbines and not wind installed capacity. This issue will become more important in the future as wind turbines with vastly larger generation capacity are beginning to arrive on the market. While this technology so far tends to be focused on offshore turbines where not only the wind flows are stronger but also the aesthetic issues are less relevant, it is possible that eventually, these bigger turbines will be installed onshore as well. Thus, future work should seek data on wind capacity as opposed to counting turbines.

Finally, while our paper has focused on wind, future work should explore if our findings carry over to the solar industry. After all, Native lands have vast solar potential but vary in solar installed capacity. It will be crucial to see if wind development crowds out solar or there are some synergies within tribal nations that allow the co-evolution of both wind and solar.
